# Reply to Pagnoni et al. Clarifying the Clinical Utility of NTAR/RGR for PAH and CTEPH. Comment on “Iancu et al. Evaluating NT-proBNP-to-Albumin (NTAR) and RDW-to-eGFR (RGR) Ratios as Biomarkers for Predicting Hospitalization Duration and Mortality in Pulmonary Arterial Hypertension (PAH) and Chronic Thromboembolic Pulmonary Hypertension (CTEPH). *Diagnostics* 2025, *15*, 2126”

**DOI:** 10.3390/diagnostics16010056

**Published:** 2025-12-23

**Authors:** Dragos Gabriel Iancu, Liviu Cristescu, Razvan Gheorghita Mares, Andreea Varga, Ioan Tilea

**Affiliations:** 1 Doctoral School, George Emil Palade University of Medicine, Pharmacy, Science, and Technology of Targu Mures, 540142 Targu Mures, Romania; dragos-gabriel.iancu@umfst.ro; 2 Faculty of Medicine, George Emil Palade University of Medicine, Pharmacy, Science, and Technology of Targu Mures, 540142 Targu Mures, Romania; razvan.mares@umfst.ro (R.G.M.); ioan.tilea@umfst.ro (I.T.); 3 Faculty of Medicine in English, George Emil Palade University of Medicine, Pharmacy, Science, and Technology of Targu Mures, 540142 Targu Mures, Romania; andreea.varga@umfst.ro

We thank Pagnoni et al. for their constructive remarks [1]. Our study was explicitly framed as an exploratory evaluation of two composite indices (NTAR and RGR). As stated in the Materials and Methods Section, “These exploratory indices were benchmarked against established prognostic markers to determine their incremental predictive value.” This sentence captures our intended scope: exploratory benchmarking of discrimination rather than a full incremental-utility program [2].

This Reply clarifies the terminology and scope and outlines the prospective validation; it does not alter the article’s conclusions.

(1) On the meaning of “ELOS”.

In our paper, ELOS denotes Extended Length of Stay (>7 days), anchored to the REVEAL mean LOS (~6.9 days) [3] and defined in Methods §2.6 (“LOS, ELOS, and Mortality Assessment”); it does not refer to one-year overall survival [2].

(2) Unadjusted vs. multivariable models.

Our analyses were limited to unadjusted models, as stated in the manuscript: “Analyzed data are presented raw and unadjusted for confounders.” This decision reflects the low number of events for both mortality endpoints reported in Table 1 (in-hospital deaths: PAH 8, CTEPH 4; 3-month deaths: PAH 7, CTEPH 3). Under standard events-per-variable (EPV) considerations (EPV = events/k), any reasonable multivariable specification would be at high risk of overfitting [4,5]. For example, with k = 5 prespecified predictors (biomarker, age, sex, WHO-FC, eGFR), EPV would be 1.6 (PAH in-hospital), 0.8 (CTEPH in-hospital), 1.4 (PAH 3-month), and 0.6 (CTEPH 3-month). Even with k = 3, EPV remains ≤2.7 across strata. Consistent with the exploratory benchmarking scope of the study, we therefore prioritized discrimination (ROC/AUC with 95% CIs; DeLong comparisons) [6] and basic calibration (Hosmer–Lemeshow) [7]. In the prospective multicenter extension now underway, we will report covariate-adjusted effects for all endpoints, handle repeated admissions (cluster-robust/GEE), and perform full internal validation.

Event counts are reported in Table 1, from which the EPV examples above are derived.

(3) Covariate-adjusted effects.

We agree that adjusted estimates (age, sex, WHO-FC, therapy, renal function, comorbidities) are informative. The follow-up study we are preparing will pre-specify covariates and report adjusted effects alongside discrimination and calibration.

(4) Repeated admissions/clustering.

Our unit of analysis was the admission, by design, because LOS/ELOS are admission-level endpoints. We acknowledge intra-patient dependence and will use cluster-robust (Huber–White) standard errors and/or GEE in the prospective extension [8,9].

(5) Calibration, small events, and bootstrap.

Given the low number of deaths, we kept internal validation simple (Hosmer–Lemeshow) to avoid optimism from complex models at low event counts [7]. The extension will add bootstrap optimism correction, calibration slope/intercept, and plots.

(6) Incremental value and collinearity.

Consistent with our exploratory scope (see Methods quotation above), we operationalized “incremental predictive value” via benchmarking discrimination: AUC comparisons (DeLong) of NTAR/RGR vs. established markers (albumin, NLR, eGFR, Log NT-proBNP). Because NTAR mathematically involves NT-proBNP and albumin, including all three in the same model would invite collinearity; therefore, we compared their AUCs head-to-head. Formal incremental-value metrics (NRI/IDI, likelihood-ratio tests) are planned for the follow-up validation [10].

Beyond noting ROC/DeLong, in CTEPH–ELOS we report AUCs with 95% CIs and model calibration: Log NT-proBNP 0.748 (0.664–0.821) and NTAR 0.743 (0.658–0.817), both significantly higher than albumin, NLR, eGFR, and RGR (DeLong *p* < 0.001), with no significant difference between Log NT-proBNP and NTAR [6]. These pairwise comparisons are illustrated in Figure 1b. Corresponding Youden thresholds and Hosmer–Lemeshow *p*-values were NTAR > 3.09 (HL *p* = 0.979), Log NT-proBNP > 3.47 (HL *p* = 0.510), albumin ≤ 3.95 (HL *p* = 0.012), eGFR ≤ 60 (HL *p* = 0.766), NLR > 2.77 (HL *p* = 0.667), RGR > 0.58 [7]. Univariable effect sizes were consistent (NTAR OR 3.641 [1.992–6.654]; Log NT-proBNP OR 3.947 [2.085–7.471]), and continuous-scale coherence was supported by Spearman correlations with LOS in CTEPH (Log NT-proBNP r = 0.48; NTAR r = 0.46). Collectively, these findings indicate that NTAR compacts the prognostic information conveyed by NT-proBNP and albumin and performs comparably to Log NT-proBNP for ELOS in CTEPH, while outperforming other comparators; formal NRI/IDI and Decision Curve Analysis are planned within the prospective validation.

(7) Construction and units of NTAR/RGR.

aFor clarity: RGR = RDW-SD (fL)/eGFR (mL/min/1.73 m^2^); NTAR = log10[NT-proBNP (pg/mL) / albumin (g/dL)]. These yield dimensionless, scale-robust indices under standard laboratory units and reduce skew (log transform for NT-proBNP) [11].

(8) Decision Curve Analysis (DCA).

We agree that DCA is valuable once actionable threshold probabilities and care pathways are pre-specified. As our published work is exploratory and admission time- and discrimination-focused, DCA will be performed in the prospective validation, aligned with discharge planning and early readmission-prevention pathways [12].

(9) Integration with hemodynamics/imaging and BPA response.

We concur on the importance of anchoring biomarkers to mechanisms. Uniform invasive hemodynamics and BPA trajectories were not available at admission for all cases; these will be integrated in the upcoming multicenter work.

(10) Looking ahead—what is already underway.

We are preparing a prospective, multicenter extension that will: (a) include prespecified covariates and cut-offs; (b) handle repeated admissions via cluster-robust standard errors (SEs) and/or GEE; (c) report calibration with bootstrap; (d) quantify incremental value using NRI/IDI and likelihood-ratio tests (LR tests) over NT-proBNP/albumin; and (e) include Decision Curve Analysis for net clinical benefit in discharge planning. (f) apply penalized logistic regression (e.g., Firth or ridge) if events-per-variable remain limited [13,14,15].

This work will be enabled by PHYRE-RO—the Romanian National Registry for Pulmonary Hypertension—which is being established to enroll all PH centers in Romania, providing the scale and data uniformity required for robust external validation and clinical-utility analyses. PHYRE-RO is being established with national center participation and unified data-governance; beyond routine labs it will uniformly capture invasive hemodynamics, imaging, and BPA trajectories to enable mechanism-anchored validation and decision-analytic assessment.

We appreciate the reviewers’ suggestions; they help sharpen the interpretation of our exploratory findings and directly inform the design of the forthcoming validation program, without altering the conclusions of the published article.

No changes to the published article are requested or required.
